# How much I moved: Robust biases in self-rotation perception

**DOI:** 10.3758/s13414-022-02589-x

**Published:** 2022-10-19

**Authors:** Silvia Zanchi, Luigi F. Cuturi, Giulio Sandini, Monica Gori

**Affiliations:** 1 U-VIP, Unit for Visually Impaired People, Istituto Italiano di Tecnologia (IIT), Via E. Melen 83, 16152 Genova, Italy; 2 RBCS, Robotics Brain and Cognitive Sciences, Istituto Italiano di Tecnologia (IIT), Via E. Melen 83, 16152 Genova, Italy; 3 DIBRIS Department, Università di Genova, 16145 Genova, Italy; 4 Department of Cognitive, Psychological, Pedagogical Sciences and of Cultural Studies, Università di Messina, 98122 Messina, Italy

**Keywords:** Navigation, Vestibular, Audition

## Abstract

Vestibular cues are crucial to sense the linear and angular acceleration of our head in three-dimensional space. Previous literature showed that vestibular information precociously combines with other sensory modalities, such as proprioceptive and visual, to facilitate spatial navigation. Recent studies suggest that auditory cues may improve self-motion perception as well. The present study investigated the ability to estimate passive rotational displacements with and without virtual acoustic landmarks to determine how vestibular and auditory information interact in processing self-motion information. We performed two experiments. In both, healthy participants sat on a Rotational-Translational Chair. They experienced yaw rotations along the earth-vertical axis and performed a self-motion discrimination task. Their goal was to estimate both clockwise and counterclockwise rotations’ amplitude, with no visual information available, reporting whether they felt to be rotated more or less than 45°. According to the condition, vestibular-only or audio-vestibular information was present. Between the two experiments, we manipulated the procedure of presentation of the auditory cues (passive vs. active production of sounds). We computed the point of subjective equality (PSE) as a measure of accuracy and the just noticeable difference (JND) as the precision of the estimations for each condition and direction of rotations. Results in both experiments show a strong overestimation bias of the rotations, regardless of the condition, the direction, and the sound generation conditions. Similar to previously found heading biases, this bias in rotation estimation may facilitate the perception of substantial deviations from the most relevant directions in daily navigation activities.

## Introduction

Perceiving self-motion is fundamental in maintaining orientation and performing efficient spatial navigation. When humans move through the environment, they constantly update their position and orientation, estimating movement direction, traveled distance, and trajectory. The vestibular system provides crucial information to perceive self-motion, allowing one to sense the linear and angular acceleration of the head thanks to the otoliths and the semicircular canals in the inner ears, respectively. Several studies on both humans and animals provide evidence that vestibular signals significantly contribute to spatial memory (Brandt et al., 2005; Hilliard et al., 2019), spatial orientation and navigation (Dallal et al., 2015 ; Gu, 2018 ; Karn & Cinelli, 2019 ; Xie et al., 2017) and representation of three-dimensional (3D) space (Lackner & DiZio, 2005).

In addition, the vestibular system provides essential insights into how we perceive the world in which we move. Perception usually comprises two stages of processing. First, we represent the sensory readout of the *interoceptive* and *exteroceptive* physical stimuli available in the environment; second, we interpret that representation (Seriès et al., 2009; Wei & Stocker, 2015). However, the perception could be inaccurate, leading to systematic errors in perception, otherwise known as biases. Biases can stem from morphological features of our sensory systems (e.g., Francl & Mcdermott, 2022; Gillingham & Previc, 1993; Li & Durgin, 2016). For example, the otoliths, which sense the linear acceleration of our head, are not able to distinguish the constant gravitational force from an actual linear acceleration of the head, if there are weak or absent rotational and visual cues to solve the disambiguation. In these situations, the so-called somatogravic illusion might arise, in which robust linear acceleration can be misinterpreted as a head tilt (Gillingham & Previc, 1993). Put simply, this misinterpretation of the acceleration cues leads us to think that we have our heads tilted slightly upward when it is not, likely eliciting a compensating response that works in the opposite direction (MacNeilage et al., 2007).

According to the Bayesian perspective, biases can stem also from prior knowledge (e.g., previous experience) about the world: the final stimuli of perceptual representation are indeed composed of the combination of prior knowledge and perception of physical stimuli. In this context, perceptual biases allow one to respond to environmental stimuli more efficiently than one might if perception were flawless. For instance, in vestibular perception, the Aubert effect is a well-established bias (Aubert, 1861), which leads to estimating the verticality towards the direction of the body tilt, likely due to an underestimation of the body tilt itself. A Bayesian model interprets this bias as a prior set at the most common position of the head that is 0° in the roll plane (i.e., not tilted) (De Vrijer et al., 2009). Other functional vestibular biases are found in heading perception: when attempting to estimate heading direction, lateral deviations from the straight-ahead position are over-represented to signal changes from the most common direction of movement that is the straight-ahead (Crane, 2012a; Cuturi & MacNeilage, 2013a). These findings suggest that vestibular cues are processed to obtain a functional representation of how people move through the environment, enhancing the discriminability of similar movement stimuli at the expense of representation accuracy. It nevertheless remains unclear whether spatial representation biases occurred when estimating rotational displacements in the yaw plane. Rotations in the yaw plane, which are the rotations along the earth-vertical axis, are fundamental for perceiving deviations from the straight-ahead direction during walking (e.g., sensing the veering). Previous studies on the perception of passive rotational displacements have found contrasting findings regarding the accuracy of estimation by healthy participants. Some studies reported that participants underestimate the amplitude of rotations (Blouin et al., 1995; Mergner et al., 1991). Other investigators found that participants were accurate, and there was no bias in the estimates (Siegler et al., 2000). Finally, other studies showed that participants often overestimated their passive rotational displacements (Israël et al., 1995; Ivanenko et al., 1997; Mackrous & Simoneau, 2011; Marlinsky, 1999). It is plausible that these contrasting findings derive from differences across tasks and inter-individual variability in spatial perception (Bruggeman et al., 2009; Zanchi et al., 2022). However, more investigations must be performed, considering the importance of the perception of rotational cues for locomotion. Indeed, signals coming from the semicircular canals may play an important role in perceiving complex path perception, such as curvilinear motions (Cheng & Gu, 2018).

The vestibular system interacts extensively with other sensory systems, such as visual, proprioceptive, and motor signals along the vestibular central pathway (Angelaki & Cullen, 2008). It is therefore unsurprising that the information coming from vestibular organs combines with external cues in the environment to build an efficient representation of the surrounding space and one’s movement features. For instance, previous literature has indicated that humans optimally integrate vestibular and visual information, leading to enhanced precision, for example, for heading perception (e.g., Butler et al., 2014; Fetsch et al., 2009; Gu et al., 2008). A growing body of evidence shows that vestibular signals also interact with spatialized auditory information, contributing to balance (Rumalla et al., 2015), enhancing ambulation (Karim et al., 2018), and self-motion perception (Shayman et al., 2020). Even if vision is the most accurate sense to detect spatial cues in the environment (Alais & Burr, 2004), spatialized sounds can aid spatial orientation when vision is unavailable, such as in visual impairments, or unreliable, like in the presence of fog or at night. Several studies (see Väljamäe, 2009, for a review on this topic) have revealed that moving sounds prompt vection, which is the illusion of self-motion induced by the presence of external moving cues without any true acceleration cue signaled by the vestibular system. Although auditory vection is usually weaker than the corresponding visual illusion, studies suggest that one can perceive it as rotational and translational self-motion (Riecke, 2016). Overall, these findings suggest that acoustic landmarks (i.e. external points of reference) interact with vestibular information during self-motion. Notably, vestibular information is peripherally and centrally integrated with auditory processing (Smith, 2012).

The vestibular system critically provides a functional spatial representation of our multisensory world, but it remains unclear as to what extent rotational information contributes to these functional representations of space and whether audio-vestibular interaction might modulate spatial perception. To unveil these aspects, in the present study, we aimed at investigating the perception of rotational displacements and the interaction between vestibular and auditory cues using a self-motion discrimination task. Specifically, we evaluated participants’ ability to estimate the amplitude of passive rotations in the earth-vertical yaw plane, both with and without virtual auditory landmarks. To accomplish our aims, we performed two experiments. In Experiment 1, we asked participants to estimate the amplitudes of their rotations in a discrimination task wherein vestibular and audio-vestibular trials alternated randomly. In particular, we instructed participants in audio-vestibular trials to estimate both vestibular information and auditory landmarks, which we presented before and after the rotation. The results of Experiment 1 did not clarify how participants actually used the auditory information that we made available spontaneously. Indeed, when a stimulus is available in the environment, our sensory systems likely process it differently according to whether we ourselves generate the stimulus or it is caused externally (Blakemore et al., 2000). We therefore aimed at investigating how administering active versus passive sounds may affect the processing of the auditory cues themselves. This was meant to rule out the possibility that the automatic and not controllable administration of sounds in Experiment 1 might obstruct the potential interaction between acoustic and vestibular information. In Experiment 2, we manipulated the administration of the acoustic landmarks so that they were presented before and after the rotations in correspondence of a voluntary keypad button press. Here, we focused on unveiling whether the self-generated auditory cues would affect the expected perceptual bias in self-motion perception. We hypothesized: (i) an overestimation bias in rotation perception along the same lines of heading perception literature; and (ii) an interaction between vestibular and auditory cues so that the available acoustic landmarks would have modulated displacement estimations.

Our results in both experiments revealed a robust overestimation bias in the perception of angular displacements. In other words, people perceived rotations as being wider than they were. The bias was resistant to the influence of spatialized auditory information in both experiments, showing no difference between the use of self- or externally generated acoustic landmarks. We discuss these findings considering previously found functional biases in human self-motion perception.

## Experiment 1

### Materials and method

#### Participants

Both the effect size from the strong heading biases found in Cuturi and MacNeilage’s study (2013a) and the effect size from the significant difference between the vestibular and audio-vestibular thresholds from Shayman et al. (2020) were used to calculate an a priori sample size for our experiment. G*power with alpha at .05 and 85% power yielded a sample size of 15 people. Sixteen healthy subjects (eight females, mean age: 27.1 ± 4.1 years) participated in Experiment 1. None of them were aware of the study’s aim. Subjects did not report a history of neurological, acoustic, or vestibular sensory disorders and had normal or corrected-to-normal vision. The ethics committee of the local health service (Ethics Committee, ASL 3, Genova, Italy) approved our study. It was conducted according to the guidelines of the Declaration of Helsinki (2013). All participants gave written informed consent.

#### Equipment and stimuli

**Fig. 1:**
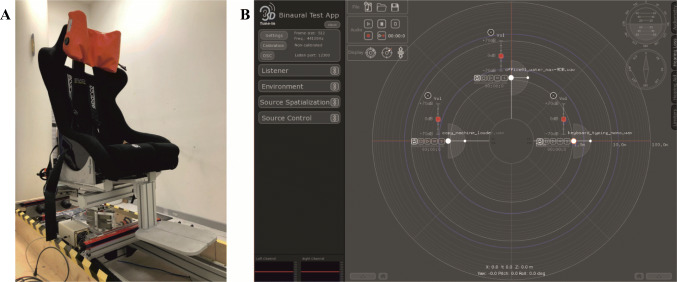
The RT-Chair (**a**). The 3D Tune-In Toolkit interface (**b**) with the representation of the three auditory landmarks (at azimuth -90°, the keyword sound, at azimuth 0° a water sound, at azimuth 90° a copy machine sound)

We administered motion stimuli using a 2-degrees-of-freedom motion platform, the Rotational-Translational Chair (RT-Chair, device internally developed by the Italian Institute of Technology, Fig. 1a; for details see Cuturi et al., 2020). In particular, the motion stimuli consisted of 3-s yaw rotations (0.33 Hz) along the earth-vertical axis, which followed a minimum jerk motion profile. The equation that describes this motion profile was previously published in Cuturi and colleagues’ study (2020). We selected 0.33-Hz motion frequency because a previous study (Shayman et al., 2020) showed the integration between auditory and vestibular cues for low-frequency stimuli (below 0.5 Hz). In the experimental procedure, rotation amplitudes ranged from 10° to 80° clockwise and from -80° to -10° counterclockwise. Peak velocities were from 6.25°/s to 50°/s, while peak accelerations ranged from 6.41°/s^2^ to 51.32°/s^2^. We controlled the motion platform using the Matlab (Matlab2017, The Mathworks, Natick, MA, USA) interface.

**Fig. 2:**
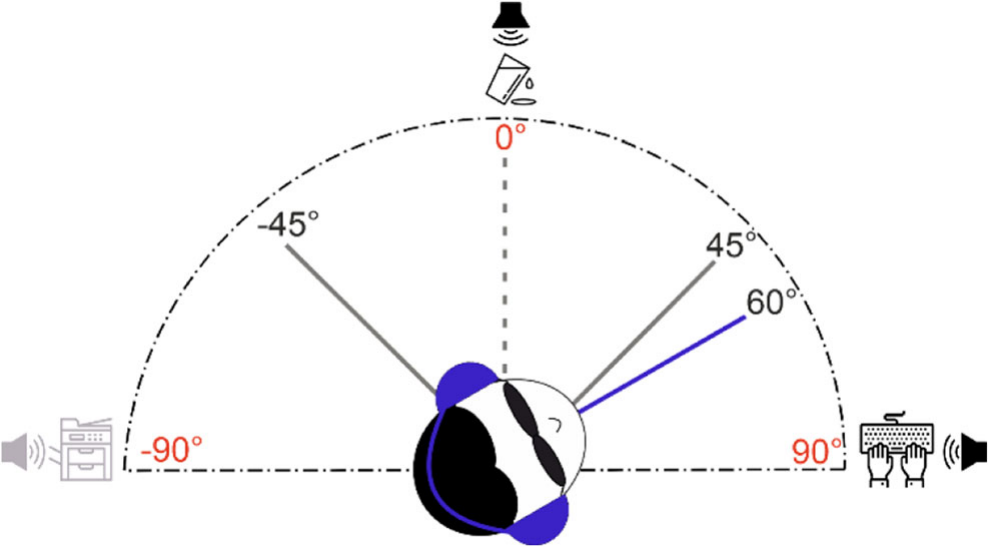
Experimental setup. The considered points of reference at azimuth -90°, 0°, and 90° are highlighted in red. In the example, the participant is rotated 60° clockwise, being closer to azimuth 90°. With clockwise rotation, the delivered auditory landmarks were the sound of the water at azimuth 0° and the sound of the keyboard at azimuth 90°, black in the picture

To deliver the spatialized auditory stimuli that worked as landmarks, we used the 3D Tune-In Toolkit (3DTI Toolkit, see Fig. 1b; (Cuevas-Rodríguez et al., 2019; Picinali et al., 2014)). This tool simulates an acoustic soundscape by using binaural spatialization, convolving monaural signals with head-related transfer functions. Using a communication protocol previously implemented by our lab, we managed the administration of the auditory stimuli by associating the 3DTI Toolkit with Matlab (Setti et al., 2021; Zanchi et al., 2021). The auditory landmarks consisted of semantic sounds (1 s each), which resemble an office environment. In particular, these were: a working copy machine, water being poured, and typing on a computer keyboard. We chose semantic sounds because of the more significant impact they have on self-motion perception compared with other sounds (Riecke, 2016). We downloaded all sounds from a royalty-free website (https://freesound.org/). Relative to the starting position at azimuth 0° (in line with participants’ nose), the copy machine sound was spatialized at azimuth -90°, the water sound at azimuth 0°, and the keyboard sound at azimuth 90° at a distance of 1.1 m (Fig. 2). Specifically, the auditory landmarks were the sound at azimuth 0° (water) and the sound at azimuth 90° (keyboard) for clockwise rotations (see example in Fig. 2), or the sound at azimuth 0° (water) and the sound at azimuth -90° (copy machine) for counterclockwise rotations. To simulate sounds fixed in the environment after the rotations, meaning to obtain landmarks to be spatialized in the exact locations at 0° and ±90° relative to the participant, sounds were presented at a position equal in amplitude to the given rotational movement but in the opposite direction. We delivered all sounds over binaural headphones (Sennheiser HD-650), used as a playback device by the toolkit.

Participants used a wireless numeric keypad to trigger movements and to provide their responses on each trial. All useful keypad buttons were shown before the experimental procedure and were made distinguishable by touch, applying a thick layer of cotton and tape.

#### Procedure

The experimental procedure was similar to the one used in our previous work (Zanchi et al., 2021). Participants were seated on the padded racing seat of the RT-Chair (Cuturi et al., 2020). Once they were comfortable, the experimenter explained the task and gave participants the headphones. Each of the participants’ heads was aligned with the RT-Chair’s rotation axis and leaned against a vacuum pillow, each time taking the shape of a participant’s head. Their forehead was held with a padded strap to the chair to reduce neck proprioceptive cues as sources of information for orientation. During the experiment, we darkened the room while participants had their eyes closed and covered by an eye mask to prevent any use of the room’s available visual information. The task of participants was to perform a self-motion discrimination task. In particular, after clockwise rotations, they had to report whether they felt closer to the point of reference at azimuth 0° (the starting point, pressing key number “4” on the left side of the keypad) or the point of reference at azimuth 90° (pressing key number “6” on the right side of the keypad). Likewise, after counterclockwise rotations, they had to report whether they felt being closer to azimuth 0° (pressing key number “6” on the right side of the keypad) or -90° (pressing key number “4” on the left side of the keypad). Participants’ responses were therefore interpreted as the perceived middle amplitude between azimuth 0° and 90°, which is a yaw rotation of 45°. For instance, if participants felt closer to azimuth 90° after a clockwise rotation, it meant they perceived a rotation wider than the middle physical amplitude between azimuth 0° and 90° (perceived rotation > 45°). Conversely, suppose participants felt closer to azimuth 0° after a clockwise rotation: this meant that they perceived a rotation smaller than the middle physical amplitude between azimuth 0° and 90° (perceived rotation < 45°). To let participants have an apparent reference of the extreme points of reference at ±90°, before the experimental session, they experienced four rotations with amplitude 90°, one for each level of the experimental design.

**Fig. 3:**
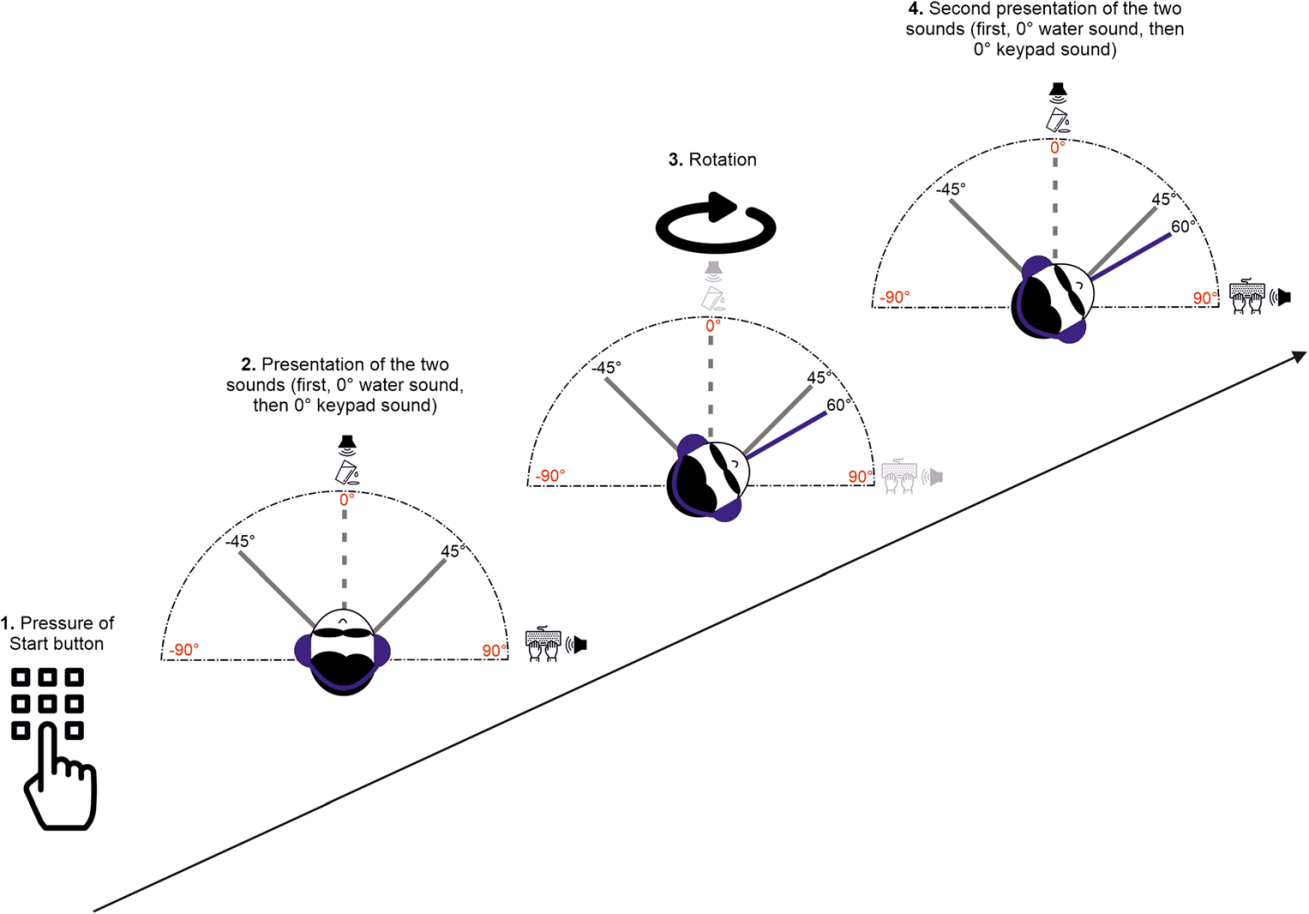
The experimental procedure in a Multisensory trial in Experiment 1. First, the participant press the start button (1); then, the two auditory landmarks were presented (2); afterwards, participant is rotated (3) and finally the two sounds are played a second time (4)

**Fig. 4:**
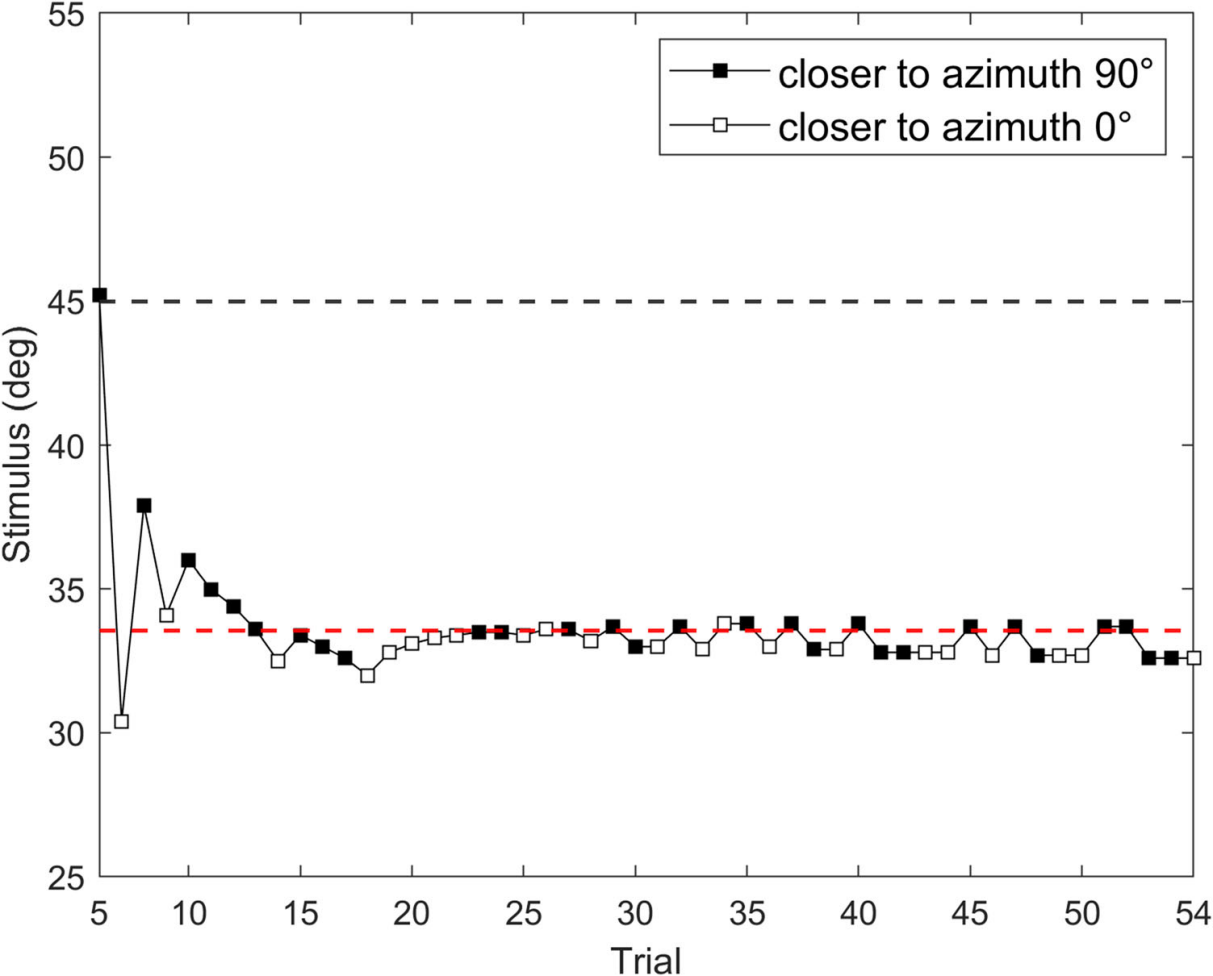
Trial history for the Psi adaptive procedure from one participant (Vestibular-only condition, clockwise rotation). Since the first four trials were training trials, only experimental ones are represented (trials from 5 to 54). The upper dashed grey line represents the investigated rotational amplitude at 45°. The lower dashed red line represents the mean of delivered motion stimuli following the Psi Procedure that approximates the point of subjective equality (PSE). White squares indicate the “closer to azimuth 0°” responses; black squares indicate the “closer to azimuth 90°” responses

Our experimental design involved testing two conditions (Vestibular-only and Multisensory, in which vestibular and auditory cues were available in the same trial) and two movement directions (clockwise and counterclockwise). On each trial, before the movement, a brief high-pitch tone through the headphones worked as a “GO” signal and was lateralized according to the direction of the forthcoming rotation (e.g., high-pitch tone in the left ear for counterclockwise rotations). After the “GO” signal sound, participants triggered the motion stimulus by pressing the start button on the keypad. In the Vestibular-only condition, participants needed to estimate their movement’s amplitude using only the vestibular cue, after either clockwise or counterclockwise rotation. In this condition, right after the pressure of the start button, participants experienced a 3-s yaw rotation, and upon finishing, they used the buttons on the keypad to give their answers. In the Multisensory condition, participants could rely on vestibular and auditory cues to estimate the rotations’ amplitude. To ensure that participants were fully aware of the positions of the sounds, we showed them a visual outline of the spatial configuration of auditory landmarks (similar to Fig. 2) before the experimental session. In this condition, after the pressure of the start button, two auditory landmarks were presented automatically and sequentially; right after the sounds, the rotation began (Fig. 3). When the rotation stopped, the auditory landmarks were again delivered automatically. During all RT-Chair rotations, white noise sound was played through headphones to mask the sounds elicited by the device. For all conditions, right after the response, participants were brought back to the start position at azimuth 0° with a reduced frequency of the just-presented stimulus (0.25 Hz). To avoid any potential aftereffects between two consecutive movements (Crane, 2012b), a 3-s time window was guaranteed between experimental motion stimuli. The conditions and rotation directions were randomized across trials for all participants. For each level of the experimental design, we assessed 54 trials, of which the first four were training trials with fixed movement magnitude. For the remaining trials, we determined rotation amplitude using the Psi adaptive procedure (Kontsevich & Tyler, 1999), which we implemented using the PAL_AMPM routine from the Palamedes toolbox (Prins & Kingdom, 2018) in Matlab (total number of trials = 216). Figure 4 shows an example of the Psi procedure for one participant and one condition (Vestibular-only, clockwise rotation). The whole experiment lasted 1 h and 30 min. We encouraged participants to take breaks at one-third and two-thirds of the experiment as a means of preventing fatigue.

#### Data analysis

**Fig. 5:**
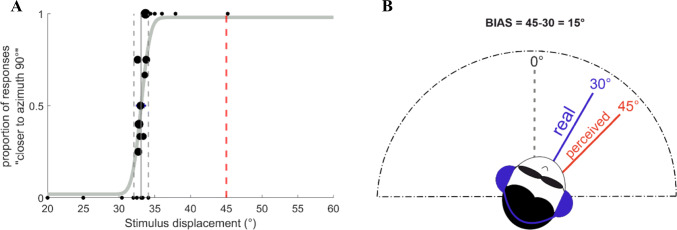
Example of psychometric fit (**a**). Individual subject data from the Vestibular-only condition with clockwise rotations are represented. The vertical dashed red line on the right indicates the unbiased estimate at 45°; the vertical solid grey line on the left indicates the point of subjective equality (PSE) (33°); the two vertical grey dashed lines on either side of the solid one represent PSE ± the just noticeable difference (JND); black points are datapoints whose size is proportional to the number of presentations for that particular stimulus displacement. Example of an overestimation bias (**b**). Here, the participant experiences a 30° clockwise rotation but perceives it as a 45° rotation, showing an overestimation bias of 15°

For clockwise rotations, we plotted the percentage of responses “I felt closer to azimuth 90°” as a function of the administered stimulus displacement (Fig. 5a). Likewise, we plotted the percentage of responses “I felt closer to azimuth 0°” for counterclockwise rotations. For each participant, condition, and direction of movement, we fitted a cumulative Gaussian to the data using the PAL_PFML_Fit routine from the Palamedes toolbox (Prins & Kingdom, 2018), which finds the best fit in a maximum likelihood sense (guess and lapse rate were fixed at 0.02). The mean provided a measure of the movement perceived as a 45° rotation (the middle amplitude between azimuth 0° and ±90°), which was considered as the point of subjective equality (PSE). We took the standard deviation of the distribution as a measurement of variability (the just noticeable difference (JND)). The JND represented the measure of the reliability of cues. Indeed, the inverse of the variability corresponds to the reliability of each cue; for example, the reliability of the vestibular cue consists of the inverse of the computed JND in the Vestibular-only condition. We calculated the error of the estimates using a non-parametric bootstrap analysis, running the function PAL_PFML_BootstrapNonParametric, generating 400 simulated data sets (Prins, 2016). We then calculated the goodness of fit by using the PAL_PFML_GoodnessOfFit function in Matlab (Prins & Kingdom, 2018).

To obtain a measure of the potential bias in amplitude estimations of rotations, we computed the difference between the unbiased amplitude of 45° and the absolute values of PSEs of each participant (bias = 45-|PSE|). Since the PSE is a measure of the movement perceived as a 45° rotation, PSE smaller than 45° meant overestimation of 45°, while PSE greater than 45° meant underestimation of 45°. Thus, we interpreted positive bias as overestimation bias (see Fig. 5b for an example) and negative as underestimation bias. Before analyzing further, we looked for outliers for each condition and the direction of rotation on our variables, namely the bias and the JND. We defined outliers as the values above the third quartile plus 1.5 times the interquartile range, and below the first quartile, minus 1.5 times the interquartile range. We excluded participants whose variables values met this definition. Specifically, regarding bias measure, this study only excluded one participant as an outlier but included 15 subjects in the final analyses. Regarding JND measure, our study excluded three subjects as outliers (13 subjects included in the final analyses). The full dataset, including outliers, is reported in the Online Supplementary Material (OSM). We verified the normality of the distribution of the variables in each condition and direction with Shapiro-Wilk tests. We performed multiple one-sample t-tests for each condition and direction to confirm whether the bias differed significantly from zero, correcting multiple tests using a Bonferroni correction. To look for differences among conditions, we conducted a two-way repeated-measures ANOVA (using the function *ezANOVA* from the *ez* package in RStudio 3.6.2, 2019), with Condition and Direction as within variables (Vestibular-only vs. Multisensory, counterclockwise vs. clockwise). We reported generalized eta squared (η^2^_G_) as effect size. We evaluated probabilities as significant when they were lower than 0.05. When in the presence of a violation of the normality assumption, we conducted the corresponding permutation tests.

### Results

**Fig. 6:**
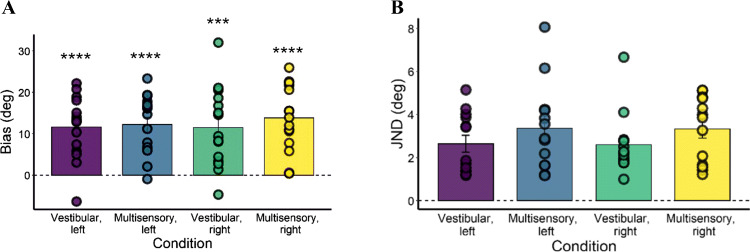
Bias (**a**) and just noticeable difference (JND) (**b**) of all participants in Experiment 1. As depicted, participants showed a strong overestimation bias (bias > 0) in all conditions, and their variability did not differ among conditions and rotation directions. Data points represent individual biases and JNDs; error bars are standard errors. **** = p < 0.0001; *** = p < 0.001

Figure 6a represents the bias mean in each condition. One-sample t-tests showed that bias was significantly different from zero in each condition and direction of movement, as shown in Table 1. Specifically, in each condition and direction, participants overestimated the rotations (bias > 0), meaning that they perceived rotations as wider than actuality. The repeated-measures ANOVA on bias values revealed no main effect of the factor Condition (*F*(1,14) = 2.511, *p* = 0.135, *η*^*2*^_*G*_ = .008), no main effect of the factor Direction (*F*(1,14) = 0.326, *p* = 0.577, *η*^*2*^_*G*_ = .002, and no interaction (*F*(1,14) = 1.155, *p* = 0.301, *η*^*2*^_*G*_ = .003).

**Table 1:** Experiment 1 one-sample t-tests on biases. T values, degrees of freedom, p values and effect size (Cohen’s *d*) reported for each condition

Condition	T values	Degrees of freedom	P values	Effect size (Cohen’s *d*)
Vestibular, left	5.92	14	0.000075	1.529
Vestibular, right	4.647	14	0.000755	1.2
Multisensory, left	6.602	14	0.000024	1.705
Multisensory, right	6.712	14	0.000020	1.733

Figure 6b depicts the JND means. Given the violation of the normality assumption for JND, we conducted a permutation ANOVA on this variable, with 5,000 permutations. It showed no main effect of the factor Condition (*p* = 0.139) and Direction (*p* = 0.907), and no interaction (*p* = 0.997).

### Experiment 1 – Discussion

Overall, results revealed a strong overestimation bias in participants’ estimation of amplitude rotations, regardless of the type of condition or the direction of rotations. Variability, measured with JND, was comparable among all conditions and directions.

In the present experiment, we provided auditory landmarks spontaneously before and after rotations. It is possible that the instant presentation of externally caused auditory cues prevented participants from actively exploring the acoustic space around them and using them for orientation. Previous studies showed that processing self-generated auditory cues is enhanced relative to passive sounds (Myers et al., 2020). It has also been shown that actively manipulating auditory stimuli might help build a spatial map of auditory cues and improve performance (Setti et al., 2018); according to a sensorimotor approach, the experience of the sensory consequences of voluntary actions allows the spatial location of any sound source to be learnt (Aytekin et al., 2008). We therefore performed a second experiment (Experiment 2), in which participants actively generated and explored auditory landmarks. In this way, we wanted to exclude the missing interaction between acoustic and vestibular cues in the results of Experiment 1 due to the inability to grasp spatial information from externally generated auditory cues.

## Experiment 2

### Materials and method

#### Participants

Since the investigated variables were the same as Experiment 1, no new a priori power analysis was required: to choose the sample size of Experiment 2, we referred again to the previous a priori power analysis, which suggested a sample size of 15. Sixteen healthy subjects (nine females, mean age: 25.6 ± 5.9 years) who did not participate in Experiment 1 participated in Experiment 2.

#### Equipment and stimuli

The vestibular stimuli delivered were the same as in Experiment 1. We previously found no difference between directions of rotation, so here we administered only clockwise rotations. To disrupt any potential habituation to clockwise rotations, we added ten counterclockwise rotations for each condition as catch trials, randomizing them across trials. Their amplitude was fixed and we chose it from among the following values: -20°,-35°,-40°,-43°,-44°,-45°,-46°,-49°,-54°, and -69°. When we administered catch trials, we told participants to perform the same task requested for the experimental trials. In the case of catch trials in the Multisensory condition, we presented the same landmarks as we did in counterclockwise rotations in Experiment 1: water sound at azimuth 0° and copy machine sound at azimuth -90°.

#### Procedure

The procedure was similar to the one used in Experiment 1. As stated in the *Equipment and stimuli* section, we delivered only clockwise experimental motion stimuli. The same discrimination task performed in Experiment 1 consisted of estimating the amplitude of rotations. The study tested two different conditions: Vestibular-only condition, in which participants needed to estimate their movement’s amplitude by exclusively using the vestibular cue, and Multisensory condition, in which they could press a button on a keypad and listen to two sounds before and after the rotation.

We wanted to evaluate the potential effect of different procedures of sound administration. In this experiment, we did not spontaneously present auditory landmarks before and after motions. For Multisensory trials, we instructed participants to press a specific keypad button (number “5” on the keypad) to listen to the sounds. Thus, before each trial, a registered voice suggested the type of condition through headphones: the Italian word for “Rotation” (*Rotazione*) indicated a Vestibular-only trial, while the Italian word for “Auditory” (*Audio*) suggested a Multisensory trial. In this way, participants knew when to press the button to explore the acoustic environment and when to press the button to only trigger the rotation. After listening to both sounds in Multisensory condition, the rotation occurred automatically. After the rotation, participants needed to press the button again to listen to the auditory landmarks. Similar to Experiment 1, the landmarks were virtually rotated after the rotation with an amplitude equal to the presented movement stimulus but in the opposite direction.

Like in Experiment 1, participants completed 54 trials in each condition, of which the first four were training trials with fixed movement magnitude. For the remaining trials, the study determined rotation amplitude using the Psi adaptive procedure (Kontsevich & Tyler, 1999) by means of the PAL_AMPM routine from the Palamedes toolbox (Prins & Kingdom, 2018) in Matlab. Responses for the ten catch trials were not included in the analyses (total number of trials = 128) as they served only to prevent habituation to clockwise movements (see above).

#### Data analysis

We performed the same fitting and analyses done in Experiment 1. Considering that there was only one direction of rotation, we conducted paired t-tests on bias and JND values to compare Vestibular-only and Multisensory conditions.

**Fig. 7:**
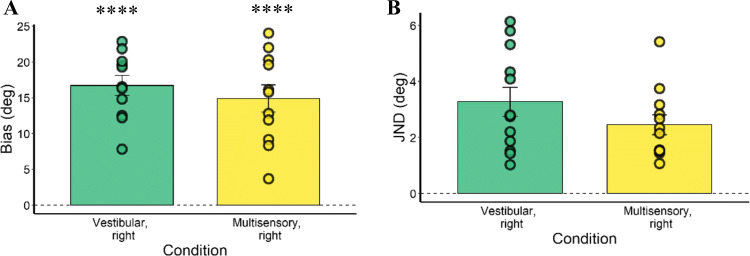
Bias (**a**) and just noticeable difference (JND) (**b**) of all participants in Experiment 2. As depicted, participants showed a strong overestimation bias (bias > 0) in both conditions, and their variability was similar between conditions. Data points represent individual biases and JNDs; error bars are standard errors. **** = p < 0.0001

For the data analysis of this experiment, we excluded one subject from the dataset because of a technical issue with the 3DTI toolkit during the experiment. In addition, the study excluded one subject owing to an extremely poor fit in one condition (successful simulations = 204 out of 400), and another because of an apparent change of response strategy during the experimental session, which influenced PSE and JND variables. We then defined outliers as done for Experiment 1. Regarding bias values, two further participants were excluded as outliers; 11 subjects were included in the final analyses. Regarding JND values (Fig. 7b), one subject was excluded as an outlier (12 subjects included in the final analyses).

### Results

Figure 7a displays the biases. One-sample t-tests showed that bias was significantly different from zero in each condition, as shown in Table 2. Similar to what we observed in Experiment 1, in each condition, participants overestimated the rotations (bias > 0). In other words, they perceived rotations as wider than they were. The paired t-test on bias revealed no significant difference between Vestibular-only and Multisensory condition (*t*(10) = -2.04, *p* = 0.07, Cohen’s *d* = 0.33).

**Table 2:** Experiment 2 one-sample t-tests on biases. T values, degrees of freedom, p values and effect size (Cohen’s *d*) reported for each condition

Condition	T values	Degrees of freedom	P values	Effect size (Cohen’s *d*)
Vestibular, right	7.782	10	0.00000749	2.346
Multisensory, right	12.071	10	0.00000028	3.64

The paired t-test on JND values revealed no significant difference between Vestibular-only and Multisensory condition (*t*(11) = 1.19, *p* = 0.26, Cohen’s *d* = 0.53).

### Experiment 2 – Discussion

Altogether, results again showed a strong overestimation bias in participants’ estimation of the amplitude of the rotation, regardless of the presence of self-generated auditory landmarks. Similarly, variability measured with JND was comparable between conditions.

## Discussion

This study investigated the ability to estimate the amplitude of passive rotational self-displacement by using only vestibular information or vestibular and auditory cues, which could be spontaneously available or self-generated by participants. The goal of Experiment 1 was to examine the human ability of self-motion estimation, with and without auditory cues. The purpose of Experiment 2 was to verify whether the expected perceptual bias in self-motion perception would be affected by the self-generated auditory cues if compared with Experiment 1’s spontaneous sound administration.

### Overestimation bias in perceived rotation

Both experiments showed that participants had a significant overestimation bias, meaning that they perceived rotations to be wider than actuality. Moreover, we observed that the auditory landmarks did not influence the bias’s amplitude. In Experiment 2, the absence of differences between Vestibular-only and Multisensory conditions revealed that participants’ manipulation of auditory cues had no effect on grasping their provided spatial information. These results are discussed considering the potential advantage for the brain of an inaccurate readout of vestibular cues.

Our results suggested a strong overestimation bias in evaluating the amplitude of whole-body yaw rotations. Past research suggests that rotational vestibular information has a crucial role in complex navigation paths of daily activities (Cuturi et al., 2021; Glasauer et al., 2002). Nonetheless, our perception of rotational signals is not flawless. Similar to our results, several previous works investigating self-motion perception found inaccuracies leading to overestimation (Israël et al., 1995; Ivanenko et al., 1997; Mackrous & Simoneau, 2011; Marlinsky, 1999). A theoretical explanation of this robust bias could rely on prior literature on heading estimation. Past studies reported an overestimation of perception of heading directions, relying on either vestibular or visual cues (Crane, 2012a; Cuturi & MacNeilage, 2013a). The similarity between vestibular and visual perceptual domains suggests a common neural mechanism underlying the bias (Crane, 2012a; Cuturi & MacNeilage, 2013a). Even though biases initially seem misleading, systematic distortions in perception can be functionally relevant in daily life and allow one to understand how the brain processes sensory information. For example, in the context of self-motion perception, overestimating a specific heading direction can be interpreted as the result of having improved sensitivity to changes relative to the direction most relevant in daily life, i.e., the straight-ahead direction, over-representing lateral directions (Cuturi & MacNeilage, 2013a). In other words, our sensory system makes us highly sensitive to directions that are perceived as uncommon to optimize the maintenance of the most common and useful ones. Arguably, such enhanced sensitivity is key to maintaining the straight-ahead direction. This means that the slightest deviation from it would be swiftly detected and corrected. For this reason, inherent perceptual biases are interpreted as the consequence of a system that attempts to optimize behavioral performance in daily life, also taking advantage of prior experiences (Cuturi, 2022; Knill & Pouget, 2004; MacNeilage et al., 2007). In this sense, the fine-tuning of the vestibular system to movements away from a preferential direction is likely to reflect the attempt to maintain efficient spatial navigation and locomotion. Similarly, the overestimation of yaw rotations that we observed in the presented experiments has likely occurred due to a higher sensitivity to changes from the most relevant starting position at azimuth 0°. It is plausible that the rotational and translational components of our movements may inform us about substantial deviations to maintain our trajectory during daily complex locomotion.

An alternative interpretation of these biases may attribute them to the cognitive effort required to estimate passive movements. Some studies reported that when the participants provide online estimates, namely during the experienced movement, the estimations were more accurate (tracking task; Ivanenko et al., 1997). The enhanced accuracy indicates that retrospective judgment of vestibular information requires more attentive resources than simply keeping track of the amplitude of motions. This is in line with the finding that a certain amount of cognitive effort is necessary to accurately use vestibular information to estimate body displacements (Yardley et al., 1999). However, this general interpretation does not clarify the direction of the bias (overestimation rather than underestimation) and fits poorly with the simple discrimination task in our experiments. In addition, Cuturi and MacNeilage (2013a) found similar perceptual biases using both heading identification and discrimination tasks, suggesting a generalized and robust perceptual mechanism that does not specifically depend on the task.

Importantly, in our study we selected 0.33-Hz rotational stimuli that are shown to be perceived with lower sensitivity by the vestibular system relative to higher frequency movements (Grabherr et al., 2008). This means that precision in discriminating low-frequency stimuli is poor. Although we did not directly investigate whether the chosen frequency affected observed results in accuracy, we can speculate the potential outcomes with other movements frequencies by considering the Bayesian perspective in interpreting biases (Crane, 2012a; Cuturi, 2022; Cuturi & MacNeilage, 2013a; Cuturi & MacNeilage, 2013b; De Vrijer et al., 2009). According to this model, the perception of a stimulus is based on combining prior information (previous knowledge and experiences) and the available sensory information encoded by our sensors (e.g., the semicircular canals in the vestibular system). In probabilistic terms, the posterior distribution representing perception of the target stimulus (e.g., a yaw rotation) is given by the probabilistic product of the prior distribution and the likelihood (i.e., the sensory input in response to the target stimulus). When sensory information is noisy and thus poorly reliable, the corresponding likelihood distribution will be more variable, thus the resultant posterior probability will be pulled toward the prior distribution. Therefore, if, in our case, the prior is a preference for the straight-ahead direction, there are reasons to believe that the poorer the sensory signal in response to the rotational stimulus, the more the perceptual bias should increase. Conversely, with increasing the frequency of the rotations, thus increasing their reliability (Grabherr et al., 2008; Valko et al., 2012), the bias should decrease or even disappear. Further studies can be formulated to test directly this hypothesis and clarify the relationship between perceptual sensitivity and accuracy in self-motion estimation for rotational stimuli.

#### Potential cortical network involved

Future studies could also investigate the possible cortical mechanisms associated with the biases of yaw rotation estimations. Indeed, the neural network that encodes self-displacement information and the contribution of vestibular signals to distance perception is not fully understood. In our work, participants estimated their traveled angular displacement. In a previous study, repetitive transcranial magnetic stimulation on the posterior parietal cortex (PPC) specifically disrupted healthy participants’ ability to reproduce previously experienced angular displacements in a path integration task (Seemungal et al., 2008). Another study with patients with acute hemisphere lesions (Kaski et al., 2016) has shown that those who reported damage to the temporo-parietal junction (TPJ) had impaired performance in estimating traveled angular distance and motion duration, but presented a healthy-like vestibular motion perception. Thus, authors suggest that TPJ may be the cortical substrate underlying the perception of vestibular complex spatial information. This evidence leads us to speculate that this cortical temporo-parietal network may underlie the self-motion estimation mechanism that leads to the overestimation biases we observed.

#### Vestibular bias and multisensory processing

We found that the magnitude of bias did not vary between conditions with and without auditory landmarks. This result indicates that static auditory cues fixed in the environment had no impact on self-motion perception accuracy. Previous literature has shown that when auditory information is provided at the same time of vestibular cues, they are optimally integrated to increase the *precision* in self-motion perception for low-frequency movements (i.e., below 0.5 Hz) (Shayman et al., 2020). Besides, the effect of the audio-vestibular combination on the *accuracy* of self-motion perception still requires investigation. According to the Maximum Likelihood Estimation (MLE) model, the optimal estimation of combined multisensory cues corresponds to the weighted average of the two cues when they have equal weights, such as equal reliability (Ernst & Banks, 2002). In contrast, if one cue is more reliable, the estimation shifts towards the former, resulting in its sensory dominance (Ernst & Banks, 2002). In the context of audio-vestibular integration (Shayman et al., 2020), vestibular and auditory information exhibit comparable reliability when the frequency of movements is between 0.2 and 0.5 Hz – the range of frequency of yaw rotations we provided in our current experiments (0.33 Hz). In contrast to Shayman and colleagues’ findings (2020), our results on precision (i.e., the measured JND) showed that the multisensory conditions did not improve precision concerning the perceptual readout of vestibular perceptual readout stimuli. This suggests that vestibular information may be more reliable than auditory, leading to the former’s predominance over the latter. Therefore, it is plausible that the higher reliability of vestibular information has resulted in a prevalence of the dominant sense (Ernst & Banks, 2002), explaining why the presence of auditory cues does not modulate the accuracy.

Importantly, the observed vestibular dominance and the difference between our study and the previous results found in Shayman et al. (2020) can be explained by one relevant feature of our experimental design. In both our experiments during Multisensory conditions, auditory landmarks were presented as static auditory cues *before and after* the rotational stimuli, but never at the same time of the vestibular stimulation. Conversely, in Shayman and colleagues’ study (2020), they provided the auditory cue during the whole duration of the displacements. With our study, we did not attempt to model our data according to the MLE as Shayman et al. (2020) did, but we aimed at studying the potential interaction (i.e., modulation of performance) between different sensory cues conveying the same spatial information. However, the absence of overlapping auditory and vestibular cues might reveal to be a limitation of our experimental design. The presence of acoustic information during motion may indeed increase the interaction between the external auditory cues and the vestibular ones. There is a need to implement future investigations to offer coherent moving acoustic signals to verify whether their reliability can increase, thus influencing the accuracy in self-motion perception.

We found a similar magnitude of bias between Experiments 1 and 2, indicating that the strength of the observed rotational biases overcomes how the auditory landmarks were presented. The rationale behind Experiment 2 relied on the idea that the association between an active motor action (the pressure of the keypad button) and its auditory consequence (auditory landmarks) may modulate using sounds for spatial orientation, improving the perception of spatial features. Previous studies showed that participants experienced enhanced processing of the sensory consequences of voluntary actions (Desantis et al., 2014; Gozli et al., 2016; Salomon et al., 2011). For instance, a previous study that used sound level discrimination and auditory detection tasks (Myers et al., 2020) showed that sensorimotor integration may improve the accuracy of the perception of self-generated sounds. Along these lines, in the rehabilitation domain, the association between active body movements and auditory stimuli allows participants to build accurate spatial representations after 30 min of training (Martolini et al., 2020). A previous study about spatial auditory memory (Setti et al., 2018) showed a significant influence of active exploration in memorizing the spatial organization of a virtual semantic acoustic scene. It is worth considering that, in the abovementioned studies about spatial auditory processing, the time of exploration was largely longer than the length in our experiments, while in Myers and colleagues’ study (2020), the enhancement in performance concerned the accuracy of perception of physical properties of sounds (loudness) that did not include their spatial representation. As one may have expected, the robust overestimation bias we have found persists regardless of the typology of presentation of auditory stimuli. Arguably, the spatial information conveyed by the vestibular system is more relevant than the spatialized auditory cues we provide here, suggesting its fundamental role for functional self-motion perception.

### Conclusion

In the current research, we demonstrated the presence of a robust overestimation bias in yaw rotations, revealing resistance to the influence of static auditory information, both automatically and actively explored. According to previously cited literature on self-motion perception, we strongly consider the discovered overestimation bias to be an efficient adaptation of the brain leading to a less accurate but more functional representation of the world.

## Data Availability

All raw data have been made publicly available on the Zenodo repository (10.5281/zenodo.6397500).
